# Proposal for a Biologic Staging System of Parkinson’s Disease

**DOI:** 10.3233/JPD-225111

**Published:** 2023-05-09

**Authors:** Lana M. Chahine, Kalpana Merchant, Andrew Siderowf, Todd Sherer, Caroline Tanner, Kenneth Marek, Tanya Simuni

**Affiliations:** a Department of Neurology, University of Pittsburgh, Pittsburgh, PA, USA; b Northwestern University Feinberg School of Medicine, Chicago, IL, USA; c Department of Neurology, University of Pennsylvania Perelman School of Medicine, Philadelphia, PA, USA; d The Michael J Fox Foundation for Parkinson’s Research, New York, NY, USA; e Department of Neurology, Weill Institute for Neurosciences, University of San Francisco, San Francisco, CA, USA; f Institute for Neurodegeneration, New Haven, CT, USA; g Department of Neurology, Northwestern University Feinberg School of Medicine, Chicago, IL, USA

**Keywords:** Parkinson’s disease, disease staging, biomarkers, alpha-synuclein

## Abstract

The Parkinson’s disease (PD) research field has seen the advent of several promising biomarkers and a deeper understanding of the clinical features of the disease from the earliest stages of pathology to manifest disease. Despite progress, a biologically based PD staging system does not exist. Such staging would be a useful framework within which to model the disease, develop and validate biomarkers, guide therapeutic development, and inform clinical trials design. We propose that the presence of aggregated neuronal α-synuclein, dopaminergic neuron dysfunction/degeneration, and clinical signs and symptoms identifies a group of individuals that have Lewy body pathology, which in early stages manifests with what is now referred to as prodromal non-motor features and later stages with the manifestations of PD and related Lewy body diseases as defined by clinical diagnostic criteria. Based on the state of the field, we herein propose a definition and staging of PD based on biology. We present the biologic basis for such a staging system and review key assumptions and evidence that support the proposed approach. We identify gaps in knowledge and delineate crucial research priorities that will inform the ultimate integrated biologic staging system for PD.

## INTRODUCTION

In the 21^st^ century, the Parkinson’s disease (PD) research field has borne witness to a shift in the conceptual framework for neurodegeneration, which is now seen as a continuum, from asymptomatic individuals who are at-risk, including carriers of relevant genetic variants, to those with mild prodromal features, all the way to established disease. With this shift has come a redefinition of the clinical diagnosis of PD [1–3], proposition of research criteria for prodromal PD [4, 5] and dementia with Lewy bodies (DLB) [6], and in both, an increasing reliance on biomarkers to detect and more objectively quantify disease stage, especially when clinical features are absent or subtle. With increasing accuracy of biomarkers has come the ability to identify groups of individuals at risk for PD and DLB or who are prodromal [7–9]—that is, those who have mild signs or symptoms within the continuum of Lewy body disorders (LBD) due to underlying neuronal α-synuclein (asyn) pathology. In turn, the possibility of preventing the disease in at-risk individuals is in reach [10–12].

We herein conceptualize a framework for a novel biologic staging system of PD. We review key assumptions as well as ambiguities and limitations in the state of the field. Based on current state of the field we delineate the foundational principles for biological staging of PD and identify gaps in knowledge, to guide the priorities for research that are needed to establish a biologic staging system of PD. This proposal applies to the clinicopathological diagnostic entities that are characterized by Lewy pathology in neuronal cell bodies and neurites and as such includes PD and DLB. We use the term PD to cover all relevant clinical syndromes. The entity of multiple system atrophy (MSA) is not included as MSA is an α-synucleinopathy defined by predominantly glial cytoplasmic inclusions and has a different biology with a different pattern of proteinopathy.

## CONCEPTUAL FRAMEWORK FOR A BIOLOGIC STAGING SYSTEM OF PD

**Fig. 1:**
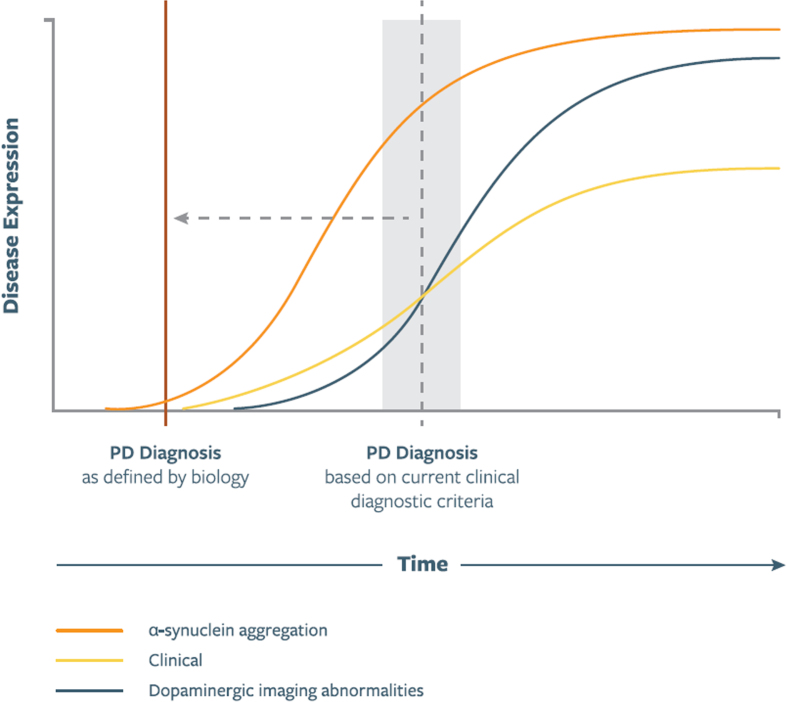
Illustration of putative relationship between asyn aggregation, dopaminergic dysfunction, and clinical manifestations as represented in a biologic staging system for PD. Curve shapes, slopes, and their temporal relationship are qualitative and hypothetical. Time of PD Diagnosis signifies diagnosis based on current clinical diagnostic criteria, which in the proposed staging system will be redefined to the time of onset of the biological changes. In future versions of the staging system these curves will be shaped by data emerging from longitudinal studies.

Five core concepts underly the proposal for a biologic staging system of PD:

∘ Neuronal asyn aggregates are indicative of a pathologic process that defines the type of neurodegenerative parkinsonian disorder to which this staging system is applicable.
∘ asyn pathology involves specific anatomic regions across the neuraxis.
∘ Pathologic asyn aggregation generally precedes dopaminergic neuron loss (Fig. 1).
∘ Dopaminergic neuron dysfunction/degeneration in the midbrain is ultimately universally present in PD.
∘ Presence of neuronal pathologic asyn aggregation, dopaminergic neuron dysfunction, and clinical signs/symptoms identifies a group of individuals that have PD pathology, which in early stages manifests with subtle non-motor and/or motor features and in later stages with a motor, cognitive, and/or other non-motor syndrome.

### Proposal for a biologic staging system of PD

We propose that a biologic staging system of PD defines PD based on the presence of neuronal pathologic asyn (S) and dopaminergic neuron dysfunction/degeneration (D). Defining the disease biologically is a departure from prior staging systems of PD that rely on clinical features. With a biologic staging system, the clinical features do not define the disease; instead, the disease is defined by the presence of pathologic asyn and dopaminergic dysfunction/degeneration. Clinical features would then be used to delineate a specific syndrome driven by the unifying biology.

asyn is the core constituent of Lewy bodies and Lewy neurites, the pathological hallmarks of PD and DLB. Although the physiological role of monomeric asyn is not fully understood, several lines of evidence indicate a role for misfolded/aggregated asyn in PD pathophysiology [13]. In light of this, accurate and sensitive measures of asyn aggregates have been pursued for decades, and an assay with high accuracy for clinical diagnosis of PD has emerged: CSF asyn seed amplification assay (SAA) [14–16]. CSF asyn SAA has > 90% sensitivity and almost 100% specificity for detecting PD and DLB [14–17], is present in prodromal cases with abnormal dopaminergic imaging [16, 18], and predicts conversion to a clinically diagnosed neurodegenerative parkinsonian syndrome in individuals at risk [18]. Based on the current state of the field, the S dimension is categorical with two strata (S- or *S*+).

Midbrain dopaminergic neuron degeneration is another core feature of clinically-manifest PD and DLB. Detected by various imaging modalities [19], it is present in all individuals with PD [2] and most individuals with DLB [20]. Dopaminergic dysfunction/degeneration begins years before onset of clinically-manifest disease (i.e., parkinsonism), but heralds its onset [8, 21, 22]. Based on the current state of the field, the D dimension is categorical with two strata (D- or *D*+). Individuals might be *S* + /D- and later advance to *S* + /D + status or may never progress to presence of dopaminergic dysfunction which would correlate to the presence of “incidental Lewy body pathology” postmortem [23–27].

Akin to the integrated staging system developed for Huntington’s disease (HD) [28], we propose that people with highly penetrant, dominantly inherited monogenic causative variants, even in absence of asyn pathology or dopaminergic dysfunction, should be assigned a stage in PD biological classification. As of today, these may include, for example, individuals carrying variants with confirmed pathogenicity in genes that cause autosomal dominant PD (*SNCA*, *VPS35*), or autosomal recessive PD (*PRKN*, *PINK-1*, and *PARK7*) [29]. The genetic research community will need to continuously reassess what variants qualify for inclusion into the staging.

Early stage PD will be based on identification of biomarkers alone. These individuals with biological anchors of *S*+ D+ will have no detectable clinical features. What is below the threshold of detection will evolve as more sensitive quantitative measures emerge. Thus, this stage will constantly be redefined. Future sensitive neurophysiological or biochemical biomarkers [30–32] may inform additional stages and lead to reclassification of some individuals. Stages that distinguish between increasing degrees of motor abnormalities will be required to provide the space within which to develop sensitive quantitative measures including digital biomarkers [33].

The S and D biomarkers allow categorical definition of negative or positive based on the current state of the field (as detailed further below). These biomarkers, once positive, will therefore remain “static” along the continuum of clinical progression. As such, clinical manifestations and their functional consequences will be used to define progression along the clinical continuum of what is currently diagnosed as PD or DLB. A progression of non-motor and motor features is seen in most (though not all) individuals with neuronal pathologic asyn and evidence of dopaminergic dysfunction/degeneration. The signs and symptoms generally align with pathology at specific anatomic structures along the neuraxis. PD is associated with a wide spectrum of non-motor manifestations that can be present from the earlier stages of the PD biological process, such as hyposmia, mild neuropsychiatric symptoms, dysautonomia or REM sleep behavior (RBD) disorder. While many of these symptoms are non-specific, their presence in the setting of biomarkers of PD will increase specificity. Later stages of the disease are marked by motor and neuropsychiatric progression and complications of therapy and the disease. While specific quantitative clinical anchors will ultimately be developed, current staging may be qualitatively conceptualized and anchored to the degree of disease-related functional impairment along the continuum of slight, mild, moderate, and severe. We advocate anchoring clinical progression to the degree of patient-perceived functional impairment, as that is a reflection of disability rather than clinical signs and symptoms. Functional impairment will need to be assessed across the spectrum of motor and non-motor disability, specifically inclusive of the cognitive domain.

Table 1 illustrates examples of how a biologic staging system would be applied; the system does not include a pathologic dimension but the hypothesized most caudal level of pathology predicted is specified for context.

**Table 1:** Illustrative Application of a Biologic Staging System for PD

Description of Individual to Which Staging System Applied	Biologic Stage	Comment
Asymptomatic carriers of SNCA pathogenic variant but with no biomarker evidence of pathology, including normal dopaminergic imaging	S_ - _D_ - _	The earliest stage will consist of individuals who are identified based on specific risk factors (highly penetrant risk variants).
		New peripheral or central biomarkers, genotype, and other environmental/behavioral risk factors could eventually inform modified staging in such individuals
Clinically asymptomatic carriers of pathogenetic variants with abnormal asyn SAA but normal dopaminergic imaging	S _ +_ D-	Pathologically these individuals may represent incidental Lewy body disease. There are reports of advanced stage pathology in these entirely asymptomatic individuals. New peripheral or central biomarkers could eventually inform modified staging in such individuals
Clinically symptomatic individuals with prodromal non-motor features (RBD, hyposmia), abnormal CSF asyn SAA, normal dopaminergic imaging, and normal motor measures	S _ +_ D_ - _	asyn SAA can detect prodromal stages of asyn pathology before nigral degeneration has occurred.
		The clinical features and biomarkers would localize pathology to Braak stage 2, caudal to the midbrain. Progression to *D* + is hypothesized and longitudinal studies examining the relationship between pathologic asyn biomarkers and onset of dopaminergic imaging will test this hypothesis.
		Peripheral asyn pathology in some individuals may be present and validation of peripheral biomarkers may inform modified staging in such individuals.
Asymptomatic individuals without detectable clinical or subclinical non-motor or motor manifestations, with abnormal CSF asyn SAA and abnormal dopaminergic imaging	S + D+	This group will constantly be redefined as sensitive methods (such as quantitative digital assessments) emerge that can detect features that are subclinical but still may be of functional relevance.
		The biomarkers would localize pathology to the midbrain (Braak stage 3) at the most caudal level.
Clinically symptomatic individuals with prodromal non-motor features (RBD, hyposmia), abnormal CSF asyn SAA, abnormal dopaminergic imaging, but normal motor measures	S _ +_ *D*_ +_	The clinical features and biomarkers would localize pathology to the midbrain (Braak stage 3) at the most caudal level.
Clinically symptomatic prodromal non-motor features and abnormal motor measures, but not qualifying for the clinical diagnosis of PD	S _ +_ *D*_ +_	Sensitive quantitative measures of motor function may inform criteria that better define stages in individuals who are manifesting motor features
Early untreated PD with abnormal asyn SAA	S+ D+	The staging system has a ceiling effect but biomarkers of disease progression in manifest PD may refine stages in more advanced disease.
Parkinsonism with normal asyn SAA but abnormal DAT	S_ - _D+	Examples include individuals with clinical features typical of PD and with pathogenic variants in LRRK2.
		There may be other biomarkers substituting for asyn or in addition to S in this group.
Advanced PD, motor fluctuations, cognitive deficits/dementia	S _ +_ D+	Biomarkers of disease progression in manifest PD may refine stages in more advanced disease.

asyn, α-synuclein; CSF, cerebrospinal fluid; D, dopaminergic neuron dysfunction/degeneration; DAT, dopamine transporter; PD, Parkinson’s disease; RBD, REM sleep behavior disorder; S, neuronal pathologic asyn; SAA, seed amplification assay.

### Comparison of PD, AD, and HD frameworks for disease classification

It is useful to compare PD staging with the classification schema of Alzheimer’s disease (AD) [34, 35]. PD is clinically and pathophysiologically more heterogeneous, with greater extent of involvement of central and peripheral nervous systems (PNS). Nevertheless, there exist conceptual similarities that can inform PD staging [36]. In the setting of well-established biomarkers of AD, the amyloid, tau, neurodegeneration or A/T/N classification staging system has been put forth based on proteinopathy-based imaging, CSF biomarkers, and structural imaging; clinical features are examined for consistency with the biomarkers [34, 35]. A key gap in the PD field is lack of a progression biomarker, a conceptual analogue of tau in AD staging [37]. Until such a biomarker is available this will constrain PD staging systems. Nevertheless, in PD, with the advent of proteinopathy-based PD biomarkers such as asyn SAA, it is possible to conceptualize the first version of a staging that is analogous to that in AD.

A biologic staging system for PD is also informed by the staging system put forth for HD [28]. It defines the disease as starting in the non-manifesting stage, includes caudate and putamen volumes as biomarkers of pathogenesis, and assigns stages according to motor and cognitive clinical abnormalities and their functional consequences.

## ELABORATION ON KEY CONCEPTS THAT UNDERLY A BIOLOGIC STAGING SYSTEM OF PD

### Categorizing PD within the continuum of neurodegeneration

It may be argued that defining discrete stages is counter to evidence that the disease process and its manifestations are a continuum. We posit that at this critical juncture, identifying finite points along the continuum of PD neurodegeneration is still necessary. Several gaps exist in the understanding of etiopathogenesis, diagnosis, prognostication, and treatment of PD pathology. Defining relative boundaries using existing knowledge provides a research framework within which to develop and refine this knowledge in cellular and animal models, observational studies in humans, and clinical trials. The current level of evidence allows for definition of stages based on clinical abnormalities and their functional consequences but, as elaborated on further below, the biomarker domains are binary at this time. Once knowledge gaps are filled with quantitative PD biomarkers, so too will additional strata be defined within the biomarker domains, which will be critical toward a model applicable across the entire disease spectrum.

Disease staging is a “classification system that produces clusters of patients who require similar treatments and have similar expected outcomes. Staging can serve as the basis for clustering clinically homogeneous patients” [38] and clinically can be used to help define treatments and assess their efficacy. Many disease staging systems are currently in use in clinical care [38]. While the ultimate goal is definition of a staging system that can translate to meaningful clinical benefit to individuals with PD and those at risk, initial iterations will primarily be a research tool, informing the research which in turn will inform staging systems of the future.

A biologic staging system, by defining discrete stages, will have applications in disease modeling, biomarker development, prediction and quantification of risk and disease progression, therapeutic targeting, and trial design. Such a staging system will allow for definition of inclusion criteria in future clinical trials based on underlying biology. Clinical trials testing agents that target pathologic asyn may incorporate biomarkers of pathologic asyn for sample selection—for example, by only enrolling individuals who are *S* + . Once measures of pathologic asyn emerge that reflect disease severity, they may also be employed as outcome measures in such trials.

### Neuronal α-synuclein pathologic aggregates define PD biologically

The proposed staging system puts asyn at the front and center as a PD biomarker because it is a key feature of the hallmark Lewy pathology of PD. Converging lines of evidence implicate asyn in PD pathophysiology. αsyn aggregation is likely the downstream effect of different molecular pathways, whether synaptic, lysosomal, or mitochondrial dysfunction, abnormal vesicular trafficking, neuroinflammation, or others [39]. Future molecular therapies for PD will have diverse targets, perhaps depending on disease stage. Some targets will be upstream from asyn, but with downstream effects on Lewy pathology. In such a model, asyn is incontrovertibly present, as a marker at a minimum, and integral to the pathophysiology as a possibility. With the latter statement we acknowledge gaps in knowledge related to the contribution of asyn to PD pathophysiology [39]. Accounting for this, the biologic staging system for PD does not require asyn to be pathogenic; it only requires aggregated asyn to be present in order for an individual to receive a designation of abnormal in the S domain.

### Pathologic asyn aggregation likely precedes dopaminergic neuron loss

Several lines of evidence support the assertion that pathologic asyn aggregation precedes dopaminergic neuron loss [13] (Fig. 1). For example, the occurrence of Lewy bodies in older adults clinically free of parkinsonism and with intact nigral dopaminergic neuron populations [27], and onset of prodromal features specific to α-synucleinopathies (e.g., RBD) preceding dopaminergic abnormalities on neuroimaging [40]. Animal studies also suggest an early role for asyn pathology in the neurodegenerative process [41].

While evidence indicates accumulation of pathologic asyn is toxic to dopaminergic neurons, we acknowledge that this remains controversial. A relationship between extent of asyn pathology and dopaminergic neuronal cell loss based on cell counts is not consistently demonstrated [42]. The staging system thus does not require asyn to be causative of dopaminergic neuronal loss, but only specifies the temporal relationship between these two events.

### The staging system is agnostic to the localization and spread of synuclein

Extensive data indicate involvement of specific anatomic regions with asyn pathology. An emerging hypothesis is that prion-like mechanisms underlie spread of asyn pathology from one region to another [43]. There is a large body of evidence generated since the Braak hypothesis was put forth indicating a caudo-rostral spread of PD pathology [44], with initiation of pathology beginning as caudally in the neuraxis as the enteric nervous system, with spread rostrally to the CNS perhaps through the vagus nerve [43–45]. Recently, this pattern of spread has been referred to as “Body-First” or “Bottom-Up” progression of asyn pathology [46]. While the majority of neuropathologically examined cases of PD demonstrate a caudo-rostral gradient of pathology [39], especially cases that follow the typical and most common presentation of idiopathic PD, a substantial minority do not [47–50]. A second pattern of spread posits a “Brain-First” or “Top-down” progression [46]. This idea was put forth based, in part, on the observation that in some patients RBD may manifest only after onset of parkinsonism or dementia. Cases of amygdala-predominant Lewy body distribution in individuals with AD have also been observed [51]. These have been postulated to represent a distinct synucleinopathy [52].

While presence of pathologic asyn in the periphery is incontrovertible, an open question is whether the peripheral pathology begins first, follows, or occurs concurrently to, CNS involvement [27]. Human autopsy studies indicate that peripheral asyn pathology in the absence of CNS asyn pathology is extremely rare, at least among those with clinically-established disease [39, 53, 54]. But it is possible that both anterograde and retrograde spread occur [27]. One theory [55] even postulates parallel degeneration in the CNS and PNS but different region-specific functional thresholds lead to different patterns of progression of non-motor and motor prodromal symptoms [46].

Postmortem studies and *in vivo* studies on individuals across the spectrum of at-risk and prodromal, with and without abnormal biomarkers, are required to determine whether there is a PNS-only phase that would inform a PD staging system, or more broadly, how the pattern of spread of asyn could inform a PD staging system. Furthermore, mechanisms of abnormal asyn accumulation need further investigation, and whether asyn pathology results from prion-like spread remains an open question [56]. Instead, the topographical distribution of pathology may reflect involvement of selectively vulnerable neuronal populations.

In not requiring individuals to pass through each stage, the staging system does not mandate any specific sequence of spread of pathology. asyn imaging agents are critically needed to investigate asyn spread *in vivo*. The proposed staging system remains agnostic to any mechanisms of spread, and the clinical dimension is anchored to presumed anatomical regions of asyn pathology.

### Dopaminergic neuron dysfunction and degeneration is required for this staging system

The proposed staging system requires presence of dopaminergic degeneration. At the current state of the field, we do not have definitive *in vivo* measures of degeneration but there are solid data that presence of presynaptic dopaminergic dysfunction as assessed by imaging correlates with the postmortem evidence of degeneration [57, 58]. Consistently, there is ample evidence indicating that individuals with manifest parkinsonism (without dementia) but with scans without evidence of dopaminergic deficiency do not have a neurodegenerative disorder [59].

Several modalities exist to measure dopaminergic dysfunction and degeneration [19]. The most widely used is dopamine transporter (DAT) SPECT, but others include ultrasonography, VMAT2 PET, nigral free water diffusivity, and neuromelanin-sensitive MRI. Which imaging measure of dopaminergic dysfunction is most sensitive and specific in prodromal stages needs to be defined.

### Additional biomarkers will emerge and will inform biologic PD staging

**Fig. 2:**
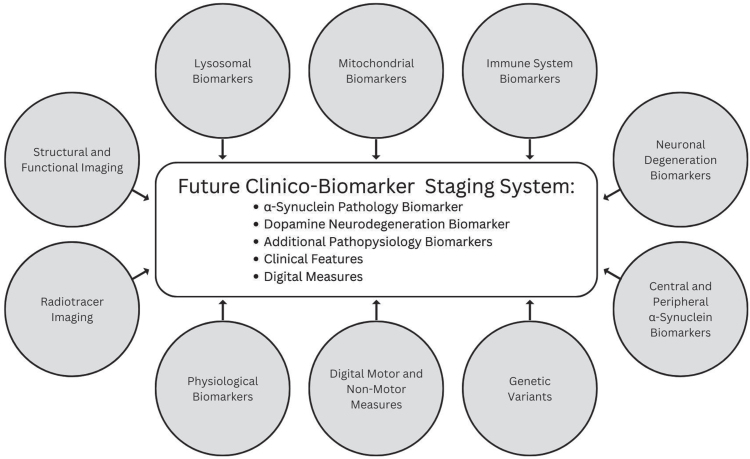
Promising categories of biomarkers that may inform future versions of a biologic staging system for PD.

Several additional promising biomarkers that represent molecular, imaging, or physiological changes underpinning PD and digital measures sensitive to subtle signs and symptoms are emerging that may be incorporated in future iterations (Fig. 2).

CSF asyn SAA is currently the most robust and reproducible method for detecting pathologic asyn [14–16], but asyn SAA is evolving and may be measurable in other tissue and fluid [14]. A robust asyn SAA in easily-accessible peripheral specimens such as skin will be an important step toward wide-spread application of a biologic staging system.

We propose a biologic staging system for PD that is anchored in the two biomarkers that most consistently reflect disease pathology and are already widely tested, reliable and have face validity. Based on the current level of evidence, these biomarkers are defined in binary categories (positive or negative), and thus remain static (once positive). While DAT binding demonstrates some variance, generally once it declines below what is expected for age and sex, it can be expected to remain below that threshold. Longitudinal progression of DAT binding can be quantitated and relates to disease severity and progression [8, 19, 21, 60–62]. However, correlations between DAT binding and clinical manifestations are modest at best [63], and DAT binding demonstrates a floor effect. Thus, there remain gaps in knowledge which do not yet allow for strata within the D dimension. Similarly, once CSF asyn SAA becomes positive, it remains positive [15, 16]. However, data indicate that kinetics of aggregation measures generated with the CSF asyn SAA do not reflect disease severity [15, 16] also necessitating a binary designation for the time being. As quantitative biomarkers of asyn, or other biomarkers that track with progression emerge, additional S strata will be added. Promising forthcoming measures include post-translationally modified forms of asyn [64, 65] and asyn derived from neuronal extracellular vesicles [66, 67].

Dopaminergic neuron dysfunction/degeneration is the core lesion of motor parkinsonism, but abnormalities in other neurotransmitter systems (noradrenergic, serotonergic, cholinergic) are implicated in PD and may be more relevant in the prodrome [68–70] and advanced disease. Future iterations of the staging system may also incorporate central and peripheral imaging of these and other neurotransmitter systems.

In the future, unbiased biomarker-based [71] or multimodal [46] data-driven approaches agnostic to candidate biomarkers or clinical features may identify or refine disease stages, but a purely unbiased data-driven approach is premature.

### The proposed staging system applies to PD and DLB

Encompassed within the proposed biologic definition of PD are several clinical syndromes which are pathologically and biologically similar, and differ only in their severity and course/progression of symptoms. These include motor predominant disease—PD as previously defined by clinical diagnostic criteria as well as dementias marked by Lewy body pathology—PD dementia and DLB. Other clinical syndromes that fall under the biologic definition of PD include RBD, and other profiles that may be defined by a composite of other signs and symptoms, including hyposmia, dysautonomia, or other neuropsychiatric manifestations—individuals who are defined as “prodromal” based on the Movement Disorders Society diagnostic criteria for prodromal PD [4, 5]. In addition, the staging system includes individuals who have biologically-defined disease and pathogenic variants that are highly penetrant for PD but are not yet manifesting any clinical syndrome.

### The proposed staging system does not incorporate multiple system atrophy

While pathologic asyn and abnormal dopaminergic imaging are present in MSA, pathophysiologically it differs from PD and DLB due to predominantly non-neuronal glial cellular structures involved, distribution of pathology, and strains of asyn implicated [72]. Importantly, aggregation kinetics of the CSF SAA for asyn is different in PD/DLB compared to MSA, and distinguishes between these disorders [14, 73, 74].

### Subjects with parkinsonism without asyn pathology likely have a yet unknown pathology and are not defined within this staging system

Studies have identified a group of patients who manifest features of typical PD, including levodopa-responsiveness and abnormal dopaminergic imaging, but lack evidence of asyn pathology with CSF SAA or on postmortem neuropathology [15, 16, 75]. These individuals would be classified as S-D+. The prototypical example are some individuals with parkinsonism associated with LRRK2 G2019S or parkin mutation [15, 16, 75]. At least two hypotheses exist regarding this observation. First is that these individuals truly do not have α-synucleinopathy. In these cases, genetic variants may lead to neuronal degeneration through other pathways, such as TDP-43- or tau-mediated neurodegeneration. Further investigation of pathological findings in individuals with clinical features of PD but without evidence of pathologic asyn is critically needed; future staging systems may incorporate additional proteinopathy-based biomarkers separate from asyn. The second possibility is that the strain of asyn aggregates in LRRK2-mediated PD pathophysiology is not being detected (false negative assay). This is a key gap in knowledge that will be filled as assays for different forms of pathologic asyn are developed, which may inform future strata of the S domain [64].

### A biologic PD staging system accelerates therapeutic development

If the continuum of PD pathology is considered on a population level, how such a staging system can be applied to prevention strategies can be envisioned [76]. Primary prevention would target individuals who are at-risk, such as asymptomatic carriers of pathogenic PD risk variants, but who do not demonstrate biomarker evidence of abnormal asyn, to prevent onset of pathology. It is likely that in the short-term, most prevention strategies will be secondary prevention, for example, among those who are *S* + D-, prevention of onset of dopaminergic degeneration. In those who have more advanced pathology, secondary prevention would entail halting clinical manifestations at non-motor features, before motor features emerge. Biomarkers that can measure risk for and progression between each stage are perhaps the largest existing knowledge gap and are critically needed.

## FUTURE DIRECTIONS: DEVELOPMENT OF AN INTEGRATED CLINICO-BIOLOGIC STAGING SYSTEM

We have focused on discussion of the biological anchors for PD definition and early stages of progression. However, based on the current state of the field, and until quantitative biomarkers of disease severity exist that allow for a purely biomarker-based staging system, a staging system for the continuum of PD will also need to define stages based on clinical signs and symptoms and their functional consequences. Data from prospective observational cohort studies are needed to inform how to define functional anchors for these stages; until such data are available, these have to be mapped to the broad categories of slight, mild, moderate, and severe functional impairment. This approach is consistent with the AD and HD staging paradigms and places emphasis on the degree of functional impairment rather than clinical signs. There are ample data from observational and interventional studies in PD to test performance of various currently available functional scales. Ultimately, the field will need to develop novel participant reported outcomes that will be sensitive to capture functional impairment in the earliest stages of disease continuum. Current knowledge of clinical and pathologic progression allows for conceptualization of general groups of individuals that may fall into the stages. While pathologic spread of asyn caudo-rostrally provides an intuitive model on which to localize clinical features, it is acknowledged that there is marked variability and multi-dimensionality (motor and non-motor) in signs and symptoms that are detectable in the PD prodrome [46] and that even people with newly diagnosed PD based on the current criteria might lack functional impairment.

## CONCLUSION

We have proposed conceptualization of a biologic staging system for PD and reviewed core concepts that form the basis of this proposal. Several ongoing observational cohort studies are providing key insights that will inform the staging system, including the definition of later stages based on clinical manifestations and their functional consequences [77–79]. Critical to the success of these efforts will be standardization of terminology, assessments and outcome measures, and data sharing. Future versions of the staging system may incorporate different measures of central and peripheral asyn, or additional biomarkers to reflect underlying pathophysiology and co-pathology. Advances in dopaminergic imaging and digital measures will likely redefine the dopaminergic and clinical dimensions of the staging system respectively. Short-term, a biologic staging system for PD will be useful to drive research priorities to fill in gaps in knowledge and in the clinical trial setting. It is expected that iterations of a biologic staging system for PD will continue to evolve and will translate to meaningful benefits to individuals with PD and those at-risk for it.

## References

[1] Berg D , Postuma RB , Bloem B , Chan P , Dubois B , Gasser T , Goetz CG , Halliday GM , Hardy J , Lang AE , Litvan I , Marek K , Obeso J , Oertel W , Olanow CW , Poewe W , Stern M , Deuschl G (2014) Time to redefine PD? Introductory statement of the MDS Task Force on the definition of Parkinson’s disease. Mov Disord 29, 454–462. 24619848 10.1002/mds.25844 PMC4204150

[2] Postuma RB , Berg D , Stern M , Poewe W , Olanow CW , Oertel W , Obeso J , Marek K , Litvan I , Lang AE , Halliday G , Goetz CG , Gasser T , Dubois B , Chan P , Bloem BR , Adler CH , Deuschl G (2015) MDS clinical diagnostic criteria for Parkinson’s disease. Mov Disord 30, 1591–1601. 26474316 10.1002/mds.26424

[3] Berg D , Adler CH , Bloem BR , Chan P , Gasser T , Goetz CG , Halliday G , Lang AE , Lewis S , Li Y , Liepelt-Scarfone I , Litvan I , Marek K , Maetzler C , Mi T , Obeso J , Oertel W , Olanow CW , Poewe W , Rios-Romenets S , Schaffer E , Seppi K , Heim B , Slow E , Stern M , Bledsoe IO , Deuschl G , Postuma RB (2018) Movement disorder society criteria for clinically established early Parkinson’s disease. Mov Disord 33, 1643–1646. 30145841 10.1002/mds.27431

[4] Heinzel S , Berg D , Gasser T , Chen H , Yao C , Postuma RB , MDS Task Force on the Definition of Parkinson’s Disease (2019) Update of the MDS research criteria for prodromal Parkinson’s disease. Mov Disord 34, 1464–1470. 31412427 10.1002/mds.27802

[5] Berg D , Postuma RB , Adler CH , Bloem BR , Chan P , Dubois B , Gasser T , Goetz CG , Halliday G , Joseph L , Lang AE , Liepelt-Scarfone I , Litvan I , Marek K , Obeso J , Oertel W , Olanow CW , Poewe W , Stern M , Deuschl G (2015) MDS research criteria for prodromal Parkinson’s disease. Mov Disord 30, 1600–1611. 26474317 10.1002/mds.26431

[6] McKeith IG , Ferman TJ , Thomas AJ , Blanc F , Boeve BF , Fujishiro H , Kantarci K , Muscio C , O’Brien JT , Postuma RB , Aarsland D , Ballard C , Bonanni L , Donaghy P , Emre M , Galvin JE , Galasko D , Goldman JG , Gomperts SN , Honig LS , Ikeda M , Leverenz JB , Lewis SJG , Marder KS , Masellis M , Salmon DP , Taylor JP , Tsuang DW , Walker Z , Tiraboschi P (2020) Research criteria for the diagnosis of prodromal dementia with Lewy bodies. Neurology 94, 743–755. 32241955 10.1212/WNL.0000000000009323 PMC7274845

[7] Siderowf A , Jennings D , Stern M , Seibyl J , Eberly S , Oakes D , Marek K , PARS Investigators (2020) Clinical and imaging progression in the PARS Cohort: Long-term follow-up. Mov Disord 35, 1550–1557. 32657461 10.1002/mds.28139

[8] Jennings D , Siderowf A , Stern M , Seibyl J , Eberly S , Oakes D , Marek K , PARS Investigators (2017) Conversion to Parkinson disease in the PARS hyposmic and dopamine transporter-deficit prodromal cohort. JAMA Neurol 74, 933–940. 28595287 10.1001/jamaneurol.2017.0985 PMC5710321

[9] Postuma RB , Iranzo A , Hu M , Hogl B , Boeve BF , Manni R , Oertel WH , Arnulf I , Ferini-Strambi L , Puligheddu M , Antelmi E , Cochen De Cock V , Arnaldi D , Mollenhauer B , Videnovic A , Sonka K , Jung KY , Kunz D , Dauvilliers Y , Provini F , Lewis SJ , Buskova J , Pavlova M , Heidbreder A , Montplaisir JY , Santamaria J , Barber TR , Stefani A , St Louis EK , Terzaghi M , Janzen A , Leu-Semenescu S , Plazzi G , Nobili F , Sixel-Doering F , Dusek P , Bes F , Cortelli P , Ehgoetz Martens K , Gagnon JF , Gaig C , Zucconi M , Trenkwalder C , Gan-Or Z , Lo C , Rolinski M , Mahlknecht P , Holzknecht E , Boeve AR , Teigen LN , Toscano G , Mayer G , Morbelli S , Dawson B , Pelletier A (2019) Risk and predictors of dementia and parkinsonism in idiopathic REM sleep behaviour disorder: A multicentre study. Brain 142, 744–759. 30789229 10.1093/brain/awz030 PMC6391615

[10] Molsberry SA , Hughes KC , Schwarzschild MA , Ascherio A (2022) Who to enroll in Parkinson disease prevention trials? The case for composite prodromal cohorts. Neurology 99, 26–33. 35970591 10.1212/WNL.0000000000200705 PMC9259088

[11] Mirelman A , Siderowf A , Chahine L (2022) Outcome assessment in Parkinson disease prevention trials: Utility of clinical and digital measures. Neurology 99, 52–60. 35970590 10.1212/WNL.0000000000200236

[12] Macklin EA , Coffey CS , Brumm MC , Seibyl JP (2022) Statistical considerations in the design of clinical trials targeting prodromal Parkinson disease. Neurology 99, 68–75. 35970588 10.1212/WNL.0000000000200897

[13] Han D , Zheng W , Wang X , Chen Z (2020) Proteostasis of α-synuclein and its role in the pathogenesis of Parkinson’s disease. Front Cell Neurosci 14, 45. 32210767 10.3389/fncel.2020.00045 PMC7075857

[14] Bellomo G , De Luca CMG , Paoletti FP , Gaetani L , Moda F , Parnetti L (2022) α-synuclein seed amplification assays for diagnosing synucleinopathies: The way forward. Neurology 99, 195–205. 35914941 10.1212/WNL.0000000000200878

[15] Brockmann K , Quadalti C , Lerche S , Rossi M , Wurster I , Baiardi S , Roeben B , Mammana A , Zimmermann M , Hauser AK , Deuschle C , Schulte C , Waniek K , Lachmann I , Sjödin S , Brinkmalm A , Blennow K , Zetterberg H , Gasser T , Parchi P (2021) Association between CSF alpha-synuclein seeding activity and genetic status in Parkinson’s disease and dementia with Lewy bodies. Acta Neuropathol Commun 9, 175. 34717775 10.1186/s40478-021-01276-6 PMC8556894

[16] Siderowf A , Concha-Marambio L , Lafontant D-E , Farris CM , Ma Y , Urenia PA , Nguyen H , Alcalay RN , Chahine LM , Foroud T , Galasko D , Kieburtz K , Merchant K , Mollenhauer B , Poston KL , Seibyl J , Simuni T , Tanner CM , Weintraub D , Videnovic A , Choi SH , Kurth R , Caspell-Garcia C , Coffey CS , Frasier M , Oliveira LMA , Hutten SJ , Sherer T , Marek K , Soto C (2023) Assessment of heterogeneity and disease onset in the Parkinson’s Progression Markers Initiative (PPMI) cohort using the α-synuclein seed amplification assay: A cross-sectional study. medRxiv, 2023.2002.2027.23286156. 10.1016/S1474-4422(23)00109-6 PMC10627170 37059509

[17] Rossi M , Candelise N , Baiardi S , Capellari S , Giannini G , Orrù CD , Antelmi E , Mammana A , Hughson AG , Calandra-Buonaura G , Ladogana A , Plazzi G , Cortelli P , Caughey B , Parchi P (2020) Ultrasensitive RT-QuIC assay with high sensitivity and specificity for Lewybody-associated synucleinopathies. Acta Neuropathol 140, 49–62. 32342188 10.1007/s00401-020-02160-8 PMC7299922

[18] Iranzo A , Fairfoul G , Ayudhaya ACN , Serradell M , Gelpi E , Vilaseca I , Sanchez-Valle R , Gaig C , Santamaria J , Tolosa E , Riha RL , Green AJE (2021) Detection of α-synuclein in CSF by RT-QuIC in patients with isolated rapid-eye-movement sleep behaviour disorder: A longitudinal observational study. Lancet Neurol 20, 203–212. 33609478 10.1016/S1474-4422(20)30449-X

[19] Mitchell T , Lehéricy S , Chiu SY , Strafella AP , Stoessl AJ , Vaillancourt DE (2021) Emerging neuroimaging biomarkers acrossdisease stage in Parkinson disease: A review. JAMA Neurol 78, 1262–1272. 34459865 10.1001/jamaneurol.2021.1312 PMC9017381

[20] Walker Z , Jaros E , Walker RW , Lee L , Costa DC , Livingston G , Ince PG , Perry R , McKeith I , Katona CL (2007) Dementia with Lewy bodies: A comparison of clinical diagnosis, FP-CIT single photon emission computed tomography imaging and autopsy. J Neurol Neurosurg Psychiatry 78, 1176–1181. 17353255 10.1136/jnnp.2006.110122 PMC2117602

[21] Chahine LM , Brumm MC , Caspell-Garcia C , Oertel W , Mollenhauer B , Amara A , Fernandez-Arcos A , Tolosa E , Simonet C , Hogl B , Videnovic A , Hutten SJ , Tanner C , Weintraub D , Burghardt E , Coffey C , Cho HR , Kieburtz K , Poston KL , Merchant K , Galasko D , Foroud T , Siderowf A , Marek K , Simuni T , Iranzo A (2021) Dopamine transporter imaging predicts clinically-defined α-synucleinopathy in REM sleep behavior disorder. Ann Clin Transl Neurol 8, 201–212. 33321002 10.1002/acn3.51269 PMC7818144

[22] Wang C , Chen F , Li Y , Liu J (2022) Possible predictors of phenoconversion in isolated REM sleep behaviour disorder: A systematic review and meta-analysis. J Neurol Neurosurg Psychiatry 93, 395–403. 34937751 10.1136/jnnp-2021-328062

[23] Iacono D , Geraci-Erck M , Rabin ML , Adler CH , Serrano G , Beach TG , Kurlan R (2015) Parkinson disease and incidental Lewy body disease: Just a question of time? Neurology 85, 1670–1679. 26468408 10.1212/WNL.0000000000002102 PMC4653112

[24] DelleDonne A , Klos KJ , Fujishiro H , Ahmed Z , Parisi JE , Josephs KA , Frigerio R , Burnett M , Wszolek ZK , Uitti RJ , Ahlskog JE , Dickson DW (2008) Incidental Lewy body disease and preclinical Parkinson disease. Arch Neurol 65, 1074–1080. 18695057 10.1001/archneur.65.8.1074

[25] Ross GW , Abbott RD , Petrovitch H , Tanner CM , Davis DG , Nelson J , Markesbery WR , Hardman J , Masaki K , Launer L , White LR (2006) Association of olfactory dysfunction with incidental Lewy bodies. Mov Disord 21, 2062–2067. 16991138 10.1002/mds.21076

[26] Ross GW , Petrovitch H , Abbott RD , Nelson J , Markesbery W , Davis D , Hardman J , Launer L , Masaki K , Tanner CM , White LR (2004) Parkinsonian signs and substantia nigra neuron density in decendentselders without PD. Ann Neurol 56, 532–539. 15389895 10.1002/ana.20226

[27] Braak H , Del Tredici K (2017) Neuropathological staging of brainpathology in sporadic Parkinson’s disease: Separating the wheat fromthe chaff. J Parkinsons Dis 7, S71–s85. 28282810 10.3233/JPD-179001 PMC5345633

[28] Tabrizi SJ , Schobel S , Gantman EC , Mansbach A , Borowsky B , Konstantinova P , Mestre TA , Panagoulias J , Ross CA , Zauderer M , Mullin AP , Romero K , Sivakumaran S , Turner EC , Long JD , Sampaio C (2022) A biological classification of Huntington’s disease: The Integrated Staging System. Lancet Neurol 21, 632–644. 35716693 10.1016/S1474-4422(22)00120-X

[29] Day JO , Mullin S (2021) The genetics of Parkinson’s disease and implications for clinical practice. Genes 12, 1006. 34208795 10.3390/genes12071006 PMC8304082

[30] Waninger S , Berka C , Stevanovic Karic M , Korszen S , Mozley PD , Henchcliffe C , Kang Y , Hesterman J , Mangoubi T , Verma A (2020) Neurophysiological biomarkers of Parkinson’s disease. J Parkinsons Dis 10, 471–480. 32116262 10.3233/JPD-191844 PMC7242849

[31] McCarter SJ , Sandness DJ , McCarter AR , Feemster JC , Teigen LN , Timm PC , Boeve BF , Silber MH , St Louis EK (2019) REM sleep muscle activity in idiopathic REM sleep behavior disorder predicts phenoconversion. Neurology 93, e1171–e1179. 31420463 10.1212/WNL.0000000000008127 PMC6808528

[32] Heimrich KG , Lehmann T , Schlattmann P , Prell T (2021) Heart rate variability analyses in Parkinson’s disease: A systematic review and meta-analysis. Brain Sci 11, 959. 34439578 10.3390/brainsci11080959 PMC8394422

[33] Simonet C , Schrag A , Lees AJ , Noyce AJ (2021) The motor prodromes of Parkinson’s disease: From bedside observation to large-scale application. J Neurol 268, 2099–2108. 31802219 10.1007/s00415-019-09642-0 PMC8179909

[34] Jack CR Jr., Bennett DA , Blennow K , Carrillo MC , Feldman HH , Frisoni GB , Hampel H , Jagust WJ , Johnson KA , Knopman DS , Petersen RC , Scheltens P , Sperling RA , Dubois B (2016) A/T/N: An unbiased descriptive classification scheme for Alzheimer disease biomarkers. Neurology 87, 539–547. 27371494 10.1212/WNL.0000000000002923 PMC4970664

[35] Jack CR Jr, Bennett DA , Blennow K , Carrillo MC , Dunn B , Haeberlein SB , Holtzman DM , Jagust W , Jessen F , Karlawish J , Liu E , Molinuevo JL , Montine T , Phelps C , Rankin KP , Rowe CC , Scheltens P , Siemers E , Snyder HM , Sperling R (2018) NIA-AA Research Framework: Toward a biological definition of Alzheimer’s disease. Alzheimers Dement 14, 535–562. 29653606 10.1016/j.jalz.2018.02.018 PMC5958625

[36] FDA (2018) Draft Guidance for Industry on Alzheimer’s Disease: Developing Drugs for the Treatment of Early Stage Disease. https://www.fda.gov/files/drugs/published/Alzheimer%E2%80%99s-Disease—Developing-Drugs-for-Treatment-Guidance-for-Industy.pdf

[37] Groot C , Smith R , Stomrud E , Binette AP , Leuzy A , Wuestefeld A , Wisse LEM , Palmqvist S , Mattsson-Carlgren N , Janelidze S , Strandberg O , Ossenkoppele R , Hansson O (2022) Phospho-tau with subthreshold tau-PET predicts increased tau accumulation rates in amyloid-positive individuals. Brain, doi: 10.1093/brain/awac329. PMC10115173 36084009

[38] Gonnella JS , Louis DZ , Gozum MVE , Callahan CA , Barnes CA (2006) Disease Staging: Clinical Criteria. Thompson Medstat, pp. 1-876.

[39] Killinger BA , Kordower JH (2019) Spreading of alpha-synuclein - relevant or epiphenomenon? J Neurochem 150, 605–611. 31152606 10.1111/jnc.14779

[40] Iranzo A , Valldeoriola F , Lomena F , Molinuevo JL , Serradell M , Salamero M , Cot A , Ros D , Pavia J , Santamaria J , Tolosa E (2011) Serial dopamine transporter imaging of nigrostriatal function in patients with idiopathic rapid-eye-movement sleep behaviour disorder: A prospective study. Lancet Neurol 10, 797–805. 21802993 10.1016/S1474-4422(11)70152-1

[41] Kim S , Kwon SH , Kam TI , Panicker N , Karuppagounder SS , Lee S , Lee JH , Kim WR , Kook M , Foss CA , Shen C , Lee H , Kulkarni S , Pasricha PJ , Lee G , Pomper MG , Dawson VL , Dawson TM , Ko HS (2019) Transneuronal propagation of pathologic α-synuclein from the gut to the brain models Parkinson’s disease. Neuron 103, 627–641.e627. 31255487 10.1016/j.neuron.2019.05.035 PMC6706297

[42] Milber JM , Noorigian JV , Morley JF , Petrovitch H , White L , Ross GW , Duda JE (2012) Lewy pathology is not the first sign of degeneration in vulnerable neurons in Parkinson disease. Neurology 79, 2307–2314. 23152586 10.1212/WNL.0b013e318278fe32 PMC3578379

[43] Visanji NP , Brooks PL , Hazrati LN , Lang AE (2013) The prion hypothesis in Parkinson’s disease: Braak to the future. Acta Neuropathol Commun 1, 2. 24252164 10.1186/2051-5960-1-2 PMC3776210

[44] Braak H , Del Tredici K , Rub U , de Vos RA , Jansen Steur EN , Braak E (2003) Staging of brain pathology related to sporadic Parkinson’s disease. Neurobiol Aging 24, 197–211. 12498954 10.1016/s0197-4580(02)00065-9

[45] Braak H , Rub U , Gai WP , Del Tredici K (2003) Idiopathic Parkinson’s disease: Possible routes by which vulnerable neuronal types may be subject to neuroinvasion by an unknown pathogen. J Neural Transm 110, 517–536. 12721813 10.1007/s00702-002-0808-2

[46] Berg D , Borghammer P , Fereshtehnejad SM , Heinzel S , Horsager J , Schaeffer E , Postuma RB (2021) Prodromal Parkinson disease subtypes - key to understanding heterogeneity. Nat Rev Neurol 17, 349–361. 33879872 10.1038/s41582-021-00486-9

[47] Coughlin DG , Petrovitch H , White LR , Noorigian J , Masaki KH , Ross GW , Duda JE (2019) Most cases with Lewy pathology in a population-based cohort adhere to the Braak progression pattern but ‘failure to fit’ is highly dependent on staging system applied. Parkinsonism Relat Disord 64, 124–131. 30948243 10.1016/j.parkreldis.2019.03.023 PMC6739131

[48] Rietdijk CD , Perez-Pardo P , Garssen J , van Wezel RJ , Kraneveld AD (2017) Exploring Braak’s hypothesis of Parkinson’s disease. Front Neurol 8, 37. 28243222 10.3389/fneur.2017.00037 PMC5304413

[49] Parkkinen L , Pirttila T , Alafuzoff I (2008) Applicability of current staging/categorization of alpha-synuclein pathology and their clinical relevance. Acta Neuropathol 115, 399–407. 18297293 10.1007/s00401-008-0346-6 PMC2270355

[50] Adler CH , Beach TG , Zhang N , Shill HA , Driver-Dunckley E , Caviness JN , Mehta SH , Sabbagh MN , Serrano GE , Sue LI , Belden CM , Powell J , Jacobson SA , Zamrini E , Shprecher D , Davis KJ , Dugger BN , Hentz JG (2019) Unified Staging System for Lewy body disorders: Clinicopathologic correlations and comparison to Braak staging. J Neuropathol Exp Neurol 78, 891–899. 31504679 10.1093/jnen/nlz080 PMC6751070

[51] Raunio A , Kaivola K , Tuimala J , Kero M , Oinas M , Polvikoski T , Paetau A , Tienari PJ , Myllykangas L (2019) Lewy-related pathology exhibits two anatomically and genetically distinct progression patterns: A population-based study of Finns aged 85. Acta Neuropathol 138, 771–782. 31494694 10.1007/s00401-019-02071-3 PMC6800868

[52] Uchikado H , Lin WL , DeLucia MW , Dickson DW (2006) Alzheimer disease with amygdala Lewy bodies: A distinct form of alpha-synucleinopathy. J Neuropathol Exp Neurol 65, 685–697. 16825955 10.1097/01.jnen.0000225908.90052.07 PMC5706655

[53] Beach TG , Adler CH , Sue LI , Shill HA , Driver-Dunckley E , Mehta SH , Intorcia AJ , Glass MJ , Walker JE , Arce R , Nelson CM , Serrano GE (2021) Vagus nerve and stomach synucleinopathy in Parkinson’s disease, incidental Lewy body disease, and normal elderly subjects: Evidence against the “body-first” hypothesis. J Parkinsons Dis 11, 1833–1843. 34151862 10.3233/JPD-212733 PMC10082635

[54] Beach TG , Adler CH , Sue LI , Vedders L , Lue L , White Iii CL , Akiyama H , Caviness JN , Shill HA , Sabbagh MN , Walker DG , Arizona Parkinson’s Disease Consortium (2010) Multi-organ distribution of phosphorylated alpha-synuclein histopathology in subjects with Lewy body disorders. Acta Neuropathol 119, 689–702. 20306269 10.1007/s00401-010-0664-3 PMC2866090

[55] Engelender S , Isacson O (2017) The threshold theory for Parkinson’s disease. Trends Neurosci 40, 4–14. 27894611 10.1016/j.tins.2016.10.008

[56] Surmeier DJ , Obeso JA , Halliday GM (2017) Parkinson’s disease is not simply a prion disorder. J Neurosci 37, 9799–9807. 29021297 10.1523/JNEUROSCI.1787-16.2017 PMC5637112

[57] Colloby SJ , McParland S , O’Brien JT , Attems J (2012) Neuropathological correlates of dopaminergic imaging in Alzheimer’s disease and Lewy body dementias. Brain 135, 2798–2808. 22961551 10.1093/brain/aws211

[58] Kraemmer J , Kovacs GG , Perju-Dumbrava L , Pirker S , Traub-Weidinger T , Pirker W (2014) Correlation of striatal dopamine transporter imaging with post mortem substantia nigra cell counts. Mov Disord 29, 1767–1773. 25048738 10.1002/mds.25975

[59] Marek K , Seibyl J , Eberly S , Oakes D , Shoulson I , Lang AE , Hyson C , Jennings D , Parkinson Study Group PRECEPT Investigators (2014) Longitudinal follow-up of SWEDD subjects in the PRECEPT Study. Neurology 82, 1791–1797. 24759846 10.1212/WNL.0000000000000424 PMC4035714

[60] Chahine LM , Siderowf A , Barnes J , Seedorff N , Caspell-Garcia C , Simuni T , Coffey CS , Galasko D , Mollenhauer B , Arnedo V , Daegele N , Frasier M , Tanner C , Kieburtz K , Marek K , The Parkinson’s Progression Markers Initiative (2019) Predicting progression in Parkinson’s disease using baseline and 1-year change measures. J Parkinsons Dis 9, 665–679. 31450510 10.3233/JPD-181518 PMC6839498

[61] Burciu RG , Ofori E , Archer DB , Wu SS , Pasternak O , McFarland NR , Okun MS , Vaillancourt DE (2017) Progression marker of Parkinson’s disease: A 4-year multi-site imaging study. Brain 140, 2183–2192. 28899020 10.1093/brain/awx146 PMC6057495

[62] Kaasinen V , Vahlberg T (2017) Striatal dopamine in Parkinson disease: A meta-analysis of imaging studies. Ann Neurol 82, 873–882. 29165839 10.1002/ana.25103

[63] Simuni T , Siderowf A , Lasch S , Coffey CS , Caspell-Garcia C , Jennings D , Tanner CM , Trojanowski JQ , Shaw LM , Seibyl J , Schuff N , Singleton A , Kieburtz K , Toga AW , Mollenhauer B , Galasko D , Chahine LM , Weintraub D , Foroud T , Tosun D , Poston K , Arnedo V , Frasier M , Sherer T , Chowdhury S , Marek K , Parkinson’s Progression Marker Initiative (2018) Longitudinal change of clinical and biological measures in early Parkinson’s disease: Parkinson’s Progression Marker Initiative cohort. Mov Disord 33, 771–782. 29572948 10.1002/mds.27361 PMC6001458

[64] Magalhães P , Lashuel HA (2022) Opportunities and challenges of alpha-synuclein as a potential biomarker for Parkinson’s disease and other synucleinopathies. NPJ Parkinsons Dis 8, 93. 35869066 10.1038/s41531-022-00357-0 PMC9307631

[65] Majbour N , Aasly J , Abdi I , Ghanem S , Erskine D , van de Berg W , El-Agnaf O (2022) Disease-associated α-synuclein aggregates as biomarkers of Parkinson disease clinical stage. Neurology 99, e2417–e2427. 36096686 10.1212/WNL.0000000000201199 PMC9687407

[66] Kluge A , Bunk J , Schaeffer E , Drobny A , Xiang W , Knacke H , Bub S , Lückstädt W , Arnold P , Lucius R , Berg D , Zunke F (2022) Detection of neuron-derived pathological α-synuclein in blood. Brain 145, 3058–3071. 35722765 10.1093/brain/awac115

[67] Niu M , Li Y , Li G , Zhou L , Luo N , Yao M , Kang W , Liu J (2020) Alongitudinal study on α-synuclein in plasma neuronalexosomes as a biomarker for Parkinson’s disease development andprogression. Eur J Neurol 27, 967–974. 32150777 10.1111/ene.14208

[68] Knudsen K , Fedorova TD , Hansen AK , Sommerauer M , Otto M , Svendsen KB , Nahimi A , Stokholm MG , Pavese N , Beier CP , Brooks DJ , Borghammer P (2018) *In-vivo* staging of pathology in REM sleep behaviour disorder: A multimodality imaging case-control study. Lancet Neurol 17, 618–628. 29866443 10.1016/S1474-4422(18)30162-5

[69] Horsager J , Andersen KB , Knudsen K , Skjærbæk C , Fedorova TD , Okkels N , Schaeffer E , Bonkat SK , Geday J , Otto M , Sommerauer M , Danielsen EH , Bech E , Kraft J , Munk OL , Hansen SD , Pavese N , Göder R , Brooks DJ , Berg D , Borghammer P (2020) Brain-firstversus body-first Parkinson’s disease: A multimodal imagingcase-control study. Brain 143, 3077–3088. 32830221 10.1093/brain/awaa238

[70] Wile DJ , Agarwal PA , Schulzer M , Mak E , Dinelle K , Shahinfard E , Vafai N , Hasegawa K , Zhang J , McKenzie J , Neilson N , Strongosky A , Uitti RJ , Guttman M , Zabetian CP , Ding YS , Adam M , Aasly J , Wszolek ZK , Farrer M , Sossi V , Stoessl AJ (2017) Serotonin and dopamine transporter PET changes in the premotor phase of LRRK2 parkinsonism: Cross-sectional studies. Lancet Neurol 16, 351–359. 28336296 10.1016/S1474-4422(17)30056-X PMC5477770

[71] Espay AJ , Schwarzschild MA , Tanner CM , Fernandez HH , Simon DK , Leverenz JB , Merola A , Chen-Plotkin A , Brundin P , Kauffman MA , Erro R , Kieburtz K , Woo D , Macklin EA , Standaert DG , Lang AE (2017) Biomarker-driven phenotyping in Parkinson’s disease: A translational missing link in disease-modifying clinical trials. Mov Disord 32, 319–324. 28233927 10.1002/mds.26913 PMC5359057

[72] Koga S , Sekiya H , Kondru N , Ross OA , Dickson DW (2021) Neuropathology and molecular diagnosis of synucleinopathies. Mol Neurodegener 16, 83. 34922583 10.1186/s13024-021-00501-z PMC8684287

[73] Poggiolini I , Gupta V , Lawton M , Lee S , El-Turabi A , Querejeta-Coma A , Trenkwalder C , Sixel-Doring F , Foubert-Samier A , Pavy-Le Traon A , Plazzi G , Biscarini F , Montplaisir J , Gagnon JF , Postuma RB , Antelmi E , Meissner WG , Mollenhauer B , Ben-Shlomo Y , Hu MT , Parkkinen L (2022) Diagnostic value of cerebrospinal fluid alpha-synuclein seed quantification in synucleinopathies. Brain 145, 584–595. 34894214 10.1093/brain/awab431 PMC9014737

[74] Kang UJ , Boehme AK , Fairfoul G , Shahnawaz M , Ma TC , Hutten SJ , Green A , Soto C (2019) Comparative study of cerebrospinal fluid α-synuclein seeding aggregation assays for diagnosis of Parkinson’s disease. Mov Disord 34, 536–544. 30840785 10.1002/mds.27646 PMC6519150

[75] Schneider SA , Alcalay RN (2017) Neuropathology of genetic synucleinopathies with parkinsonism: Review of the literature. Mov Disord 32, 1504–1523. 29124790 10.1002/mds.27193 PMC5726430

[76] Berg D , Crotty GF , Keavney JL , Schwarzschild MA , Simuni T , Tanner C (2022) Path to Parkinson disease prevention: Conclusion and outlook. Neurology 99, 76–83. 35970586 10.1212/WNL.0000000000200793

[77] Field JA , Boeve BF , Forsberg LK , St. Louis EK , Kraft RA , Nelson KM , Timm PC , Teigen LN , Avidan AY , Rivera A , Postuma RB , Gagnon JF , Pelletier A , Howell MJ , Schenck CH , De Kam J , Ryberg KJ , Summers RL , Bliwise D , Huddleston D , Wood-Siverio C , Videnovic A , Stauder M , Hersh S , Criswell SR , McLeland JS , Ju YE , NAPS Consortium (2020) The North American Prodromal Synucleinopathy (NAPS) Consortium: Baseline neuropsychological findings in 136 participants. Alzheimers Dement 16(Suppl 6), e044834.

[78] Jensen-Roberts S , Myers TL , Auinger P , Cannon P , Rowbotham HM , Coker D , Chanoff E , Soto J , Pawlik M , Amodeo K , Sharma S , Valdovinos B , Wilson R , Sarkar A , McDermott MP , Alcalay RN , Biglan K , Kinel D , Tanner C , Winter-Evans R , Augustine EF , Holloway RG , Dorsey ER , Schneider RB (2022) A remote longitudinal observational study of individuals at genetic risk for Parkinson disease: Baseline results. Neurol Genet 8, e200008. 35966918 10.1212/NXG.0000000000200008 PMC9372873

[79] Marek K , Chowdhury S , Siderowf A , Lasch S , Coffey CS , Caspell-Garcia C , Simuni T , Jennings D , Tanner CM , Trojanowski JQ , Shaw LM , Seibyl J , Schuff N , Singleton A , Kieburtz K , Toga AW , Mollenhauer B , Chahine LM , Weintraub D , Foroud T , Tosun-Turgut D , Poston K , Arnedo V , Frasier M , Sherer T ; Parkinson’sProgression Markers Initiative (2018) The Parkinson’s progressionmarkers initiative (PPMI) - establishing a PD biomarker cohort. Ann Clin Transl Neurol 5, 1460–1477. 30564614 10.1002/acn3.644 PMC6292383

